# Efgartigimod as Rescue Medication in a Patient with Therapy-Refractory Myasthenic Crisis

**DOI:** 10.1155/2024/9455237

**Published:** 2024-06-14

**Authors:** Omar Alhaj Omar, Norma J. Diel, Stefan T. Gerner, Anna Mück, Hagen B. Huttner, Heidrun H. Krämer-Best

**Affiliations:** Department of Neurology, Justus Liebig University, Giessen, Germany

## Abstract

Myasthenic crises (MC) are potentially life-threatening acute exacerbations of myasthenia gravis (MG) characterized by profound muscle weakness, bulbar symptoms, and potential for respiratory failure. Intravenous immunoglobulins (IVIG) and plasma exchange (PLEX) are conventional treatments for myasthenic exacerbations. Recently, new therapeutic options for generalized acetylcholine-receptor antibody positive (AchR+) MG were approved as an add-on therapy. They mainly consist of complement C5 inhibitors such as eculizumab and ravulizumab and neonatal Fc receptor antagonists such as efgartigimod with the approval of more options pending, e.g., zilucoplan and rozanolixizumab. More therapeutic options are in the pipeline. Although the data show a quick and reliable treatment response, these medications have not been studied for the therapy of myasthenic crisis. We present the case of a 57-year-old male with his first episode of generalized myasthenia gravis (MG) and positive acetylcholine-receptor antibodies (AchR+) who was transferred to our neurological intensive care unit with worsening generalized weakness, dysphagia, and respiratory distress. The crisis was triggered by pneumonia due to dysphagia. He was diagnosed with myasthenic crisis and treated with intravenous pyridostigmine, plasmapheresis (PLEX), and continued prednisone. Initial improvement was followed by deterioration, requiring readmission and additional PLEX. After a further decline, efgartigimod was administered, leading to significant improvement within 48 hours, as evidenced by reduced MG-ADL and QMG scores. The patient continued to improve and was stable enough for transfer to a rehabilitation facility. This case illustrates the potential of efgartigimod as a novel treatment for refractory myasthenic crises.

## 1. Introduction

Myasthenic crisis is the most severe manifestation of MG. It is a life-threatening condition defined by respiratory failure due to muscle weakness. This requires immediate medical intervention, often involving mechanical ventilation and intensive care support. In recent years, new therapeutic options have emerged to manage MG more effectively [1–3]. One such promising treatment is efgartigimod, a first-in-class neonatal Fc receptor (FcRn) antagonist. Efgartigimod works by reducing the levels of pathogenic IgG antibodies that contribute to the disease [4]. By interfering with the recycling of IgG, efgartigimod lowers the overall antibody load, thereby mitigating the immune attack on acetylcholine receptors. Here, we present a case report of a patient with a therapy-refractory myasthenic crisis after two cycles consisting of seven sessions of PLEX each, who was successfully treated with efgartigimod alpha.

## 2. Case Presentation

A 57-year-old male with first manifestation of generalized myasthenia gravis (MG) and positive acetylcholine-receptor antibodies (AchR+) was transferred from another neurological hospital to our neurological intensive care unit with progressive generalized weakness, dysphagia, and respiratory distress. He had been taking oral pyridostigmine (dose: 600 mg/day) and prednisone (dose: 20 mg/day) and was admitted to our specialized center due to worsening symptoms over the past three days. The trigger for the myasthenic crisis was pneumonia resulting from dysphagia.

His medical history includes a mechanical heart valve, depression, anxiety disorder, chronic somatoform pain disorder, arterial hypertension, and diabetes mellitus with polyneuropathy.

### 2.1. Clinical Findings

Upon admission, the patient's condition was critical. He exhibited severe dysphagia, nasal flaring, and intercostal muscle retractions, indicative of acute respiratory muscle weakness. Objective assessments using the Quantitative Myasthenia Gravis (QMG) score and the Myasthenia Gravis Activities of Daily Living (MG-ADL) questionnaire revealed scores of 29/39 and 21/24, respectively, reflecting severe functional impairment. AchR antibodies were detected with a titer of >8.00 nmol/l.

### 2.2. Diagnostic, Therapeutic, and Clinical Course

The patient was diagnosed with myasthenic crisis based on clinical presentation and serological markers. Oral therapy with pyridostigmine was switched to intravenous therapy (dose: 0.85 mg/h, equals 612 mg of oral pyridostigmine per 24 hours). Seven sessions of PLEX were performed. After an initial improvement, he was transferred to our intermediate care ward, and pyridostigmine therapy was transitioned back to oral application. Oral prednisolone at 20 mg was continued. Immunosuppressant therapy was established with azathioprine. However, despite these measures, his respiratory status and swallowing began to deteriorate two weeks after the last PLEX, with an MG-ADL score of 20/24 and a QMG score of 26/39. This prompted admission back to the neurological intensive care unit and an additional seven sessions of PLEX were performed. Oral therapy with pyridostigmine was once again switched to intravenous therapy (dose: 0.85 mg/h, equals 612 mg of oral pyridostigmine per 24 hours). However, three days after the last PLEX, symptoms started to deteriorate again with increasing dysphagia. Diagnostic work-up excluded a neoplasia or an infectious trigger of MG. After obtaining informed consent and discussing potential risks and benefits with the patient, the patient was treated with efgartigimod at a dose of 10 mg/kg, while intravenous pyridostigmine, oral prednisolone, and azathioprine were continued. The efgartigimod cycle was completed with a total of four infusions.

### 2.3. Follow-Up and Outcomes

Within 48 hours of efgartigimod alpha infusion, the patient showed significant clinical improvement evidenced by improved muscle strength and reduction in MG-ADL and QMG scores to 5/24 and 15/39, respectively. Intravenous pyridostigmine was switched to oral medication. The improvement continued over the next few weeks. His MG-ADL score improved to 3/24 and his QMG score improved to 11/39 within 48 hours of the second efgartigimod alpha infusion. The patient was transferred to a rehabilitation facility after the completion of the efgartigimod cycle in a stable clinical condition (ADL: 3/24; OMG: 11/39). The patient's response to efgartigimod underscores its potential as a novel therapeutic option in the management of myasthenic crises, particularly in cases of refractory to conventional treatments. This case highlights the need for continued exploration of new therapeutic avenues in the management of MC, especially in the context of refractory disease presentations.

## 3. Discussion

Myasthenic crises are rare but life-threatening complications of MG [5, 6]. Treatment usually includes IVIG and PLEX to lower antibody levels and modulate immune responses [7, 8]. A notable disadvantage of IVIG therapy is its delayed onset of action, typically occurring within 2–4 weeks, with the peak response observed around 20 days. In addition, the high production costs and the scarcity of volunteer blood donors limit its availability, given its widespread use for various indications, such as chronic inflammatory demyelinating polyneuropathy (CIDP) and hematological conditions. Without concomitant immunotherapy with PLEX, its clinical effect is limited to a few weeks. In recent years, the development of monoclonal antibodies targeting the neonatal Fc receptor (FcRn) has emerged as a promising and effective therapeutic option for the treatment of autoimmune diseases, including MG [4, 9]. Efgartigimod alpha, by blocking FcRn, reduces the recycling of pathogenic autoantibodies, leading to clinical improvement in patients with autoimmune diseases such as MG and stiff-person syndrome (SPS) [10]. This case report highlights a successful outcome with efgartigimod alpha in a patient who had a myasthenic crisis refractory to conventional therapies. We chose efgartigimod as the escalation treatment since the patient responded well to PLEX, which acts similarly to efgartigimod, but the symptoms reoccurred within short periods of time rendering the PLEX insufficient in the management of the myasthenic exacerbation. Since the patient was diagnosed with generalized AchR + MG, the treatment can be considered as on-label.

## 4. Conclusion

Intravenous efgartigimod alpha demonstrated rapid and significant clinical improvement in a patient with refractory myasthenic crisis. This case report suggests that anti-FcRn monoclonal antibodies may offer a promising therapeutic option not only for the escalation treatment of generalized AchR + MG with high disease activity but also for severe, refractory cases of myasthenic crisis. Further studies and clinical trials are warranted to assess the long-term benefits and safety.
